# Aqueous extract of *Peristrophe bivalvis* (L.) Merr. leaf reversed the detrimental effects of nitric oxide synthase inhibitor on blood lipid profile and glucose level

**DOI:** 10.1371/journal.pone.0308338

**Published:** 2024-09-06

**Authors:** Esther Oluwasola Aluko, Ubong Edem David, Abodunrin Adebayo Ojetola, Adesoji Adedipe Fasanmade

**Affiliations:** 1 Physiology Department, Faculty of Basic Medical Sciences, University of Uyo, Uyo, Akwa-Ibom State, Nigeria; 2 Physiology Unit, Ajayi Crowther University, Oyo, Oyo State, Nigeria; 3 Department of Physiology, Faculty of Basic Medical Sciences, Adeleke University, Ede, Osun State, Nigeria; 4 Department of Physiology, Faculty of Basic Medical Sciences, University of Ibadan, Ibadan, Oyo State, Nigeria; Georgia State University, UNITED STATES OF AMERICA

## Abstract

There is evidence that nitric oxide (NO) modulates the metabolism of glucose and lipid, and some antihypertensive medications have been shown to affect glucose and lipid metabolism. Peristrophe bivalvis is a medicinal plant that has been shown to have antihypertensive properties. The study investigated the effect of aqueous extract of Peristrophe bivalvis leaf (APB) on fasting blood glucose level (FBG) and lipid profile in rats pretreated with nitro-L-arginine methyl ester (L-NAME). Male Wistar rats (150–170 g, n=30) were randomly divided into two groups: control (CT, n=5) and L-NAME pretreated (n=25). CT received 5 mL/kg of distilled water [DW]) while L-NAME pretreated group received 60 mg/kg of L-NAME (L-NAME60) for eight weeks. After eight weeks, the L-NAME pretreated group was randomly subdivided into L-NAME group (LN), L-NAME recovery group (LRE), L-NAME ramipril group (LRA), and L-NAME APB group (LAPB). The groups received L-NAME60+DW, DW, L-NAME60+10 mg/kg ramipril, and L-NAME60+APB (200 mg/kg), respectively, for five weeks. Serum NO, lipid profile, cyclic guanosine monophosphate (cGMP), and insulin were measured by spectrophotometry, assay kits, and ELISA, respectively. Data were analysed using ANOVA at p < 0.05. At the eighth week, a fall in FBG and an increase in triglyceride, total cholesterol, and low-density lipoprotein cholesterol were recorded in L8 compared to CT. The same effects were also noticed in the thirteenth week in LN. However, FBG was significantly increased and lipid levels were decreased in LAPB compared to LN. A significant increase was observed in cGMP level in LAPB compared to LN. The study showed that APB corrected the hyperlipidemia and hypoglycemia caused by L-NAME, and this effect might be via the activation of cGMP.

## 1. Introduction

Nitric oxide (NO), a reactive nitrogen species, is the principal vasodilator produced by the vascular endothelium to regulate the vascular lumen diameter [1]. Nitric oxide is produced from the interaction of L-arginine with molecular oxygen, which results in the formation of L-citrulline and nitric oxide [2]. This reaction is catalyzed by nitric oxide synthase (NOS); the production of nitric oxide from L-arginine requires the use of reduced nicotinamide adenine dinucleotide phosphate hydrogen (NADPH) as a co-factor [2]. Flavin mononucleotide, tetrahydrobiopterin, and flavin adenine dinucleotide are additional co-factors needed for this reaction [3]. There are three isoforms of nitric oxide synthase: neuronal NOS (nNOS), also known as NOS 1, inducible NOS (iNOS), also known as NOS 2, and endothelial NOS (eNOS), also known as NOS 3 [4]. NOS 1 is associated with the nervous system and releases NO as a neurotransmitter. NOS 3 is located in the vascular endothelium and releases NO to regulate blood flow, while NOS 2 is mainly predominant in the body’s immune system. The biological function of nitric oxide can be blocked by compounds that inhibit the cellular uptake of L-arginine, substances that prevent cofactors availability, compounds that hinder the actions of NADPH and flavins, agents that scavenge nitric oxide, compounds that block the expression of NOS, and substances that prevent the binding of L-arginine to NOS [5]. The most frequently employed substances are those that prevent L-arginine from attaching to NOS. NO plays vital roles in the body, which include vasodilation of blood vessels to facilitate blood flow to the tissues, prevention of smooth muscle cell proliferation and migration, maintenance of endothelial cell function, inhibition of platelet aggregation, regulation of apoptosis, and prevention of neutrophil/platelet adhesion to endothelial cells [6]. Reduction in NO bioavailability has been associated with cardiovascular, renal, and metabolic disorders [7]. Nitric oxide synthase inhibition has been reported to hasten atherosclerosis formation [8], and enhance lipolysis. Inhibition of NOS has also been documented to elevate serum pro-atherogenic lipids and decrease anti-atherogenic lipid concentrations [9, 10].

Nitric oxide acts through a second messenger system by activating soluble guanylyl cyclase, which in turn converts guanosine triphosphate (GTP) to 3’,5’-cyclic guanosine monophosphate (cGMP) [11]. This acts as the second messenger to initiate nitric oxide effects. cGMP activates cGMP-dependent protein kinase, and this protein kinase brings about the resultant physiological modifications [12]. The other components of the NO-signaling circuit are also necessary for the initiation of the physiological effects of nitric oxide, and any of these components can be modulated to produce the physiological changes that nitric oxide induces [13]. The physiological effects of NO include promoting blood flow by vasodilation, serving as a neurotransmitter, enhancing cellular proliferation and apoptosis, and assisting in the immune response in the body [14]. Furthermore, nitric oxide has also been shown to influence glucose metabolism [15–18]. Skeletal muscle is the tissue with the largest glucose uptake capacity, and nitric oxide has been documented to modulate its glucose uptake. However, the reports of its role in glucose metabolism are incongruent. Some research on both humans and animals claimed that blocking NOS during exercise had no effect on the uptake of glucose by the skeletal muscle [19, 20]. In contrast, Higaki et al. [21] and Durham et al. [22] reported that nitric oxide plays a role in the uptake of glucose by skeletal muscle. They both showed that glucose uptake in the skeletal muscle at rest was enhanced by exogenous NO donors. Higaki et al. [21] and Durham et al. [22] studies revealed that nitric oxide donor reduces blood glucose level by facilitating its uptake by the skeletal muscle. Other studies [23–27] found that inhibiting nitric oxide synthase reduced the amount of glucose that was absorbed by muscle during exercise in both humans and animals. The findings of these studies showed that the inhibition of nitric oxide synthase increases blood glucose level by reducing its absorption by the skeletal muscle during exercise. The disparity in the studies above might be due to the type of exercise model used in these studies. For instance, in the Bradley et al. [24] study, the subjects were engaged in cycling exercise, while Heinonen et al. [20] positioned the right legs of the subjects in an in-house-designed leg exercise dynamometer. Negating the studies above, other studies reported that inhibition of NOS reduces blood glucose level and administration of nitric oxide donor increases blood glucose concentration [28–30].

NO has been reported to modulate the metabolism of lipids. Furthermore, the adipose tissue has been suggested as a source of NO release, and both inducible NOS (iNOS) and eNOS have been identified in the tissue [31]. Nitric oxide activates the transcription factor for low-density lipoprotein cholesterol (LDL-C) receptors and thereby facilitates the removal of LDL-C from the blood into the hepatic cells. LDL-C receptors are located on the hepatic cells and are responsible for maintaining the plasma concentration of LDL-C by removing it from the blood [31]. Goudarz and colleagues [32] reported that the administration of Nitro-L-arginine methy lester (L-NAME) resulted in a significant increase in LDL-C and triglyceride levels. In line with the study of Goudarz et al. [32], our study also observed an increase in triglyceride, total cholesterol, and LDL-C and a decrease in high-density lipoprotein cholesterol in rats administered L-NAME [9].

The use of traditional herbs as an alternative for the treatment of diseases is gaining popularity. Traditional uses of the *Peristrophe bivalvis* include the management of diabetes, hypertension, hepatitis, and tuberculosis [33]. Zhuang et al. [34] and Cheng et al. [35] reported its antihypertensive ability. Similar to this, our laboratory investigated the antihypertensive potential of various *Peristrophe bivalvis* leaf extracts and discovered that the aqueous extract demonstrated the greatest efficacy [36]. *Peristrophe bivalvis* leaf extract has antihypertensive ability; however, its effects on other physiological modalities such as blood lipid and glucose levels have not been widely reported. Antihypertensive agents have been documented to cause hyperlipidemia, glucose intolerance, and a reduction of high-density lipoproteins [37, 38]. Besides, some antihypertensive drugs have been reported to avert atherosclerosis and enhance insulin sensitivity, while others have been shown not to have any effect on either glucose or lipid metabolism [39, 40]. The present work was designed to evaluate the effect of aqueous extract of Peristrophe bivalvis (APB) on fasting blood level and lipid profile in rats pretreated with nitric oxide synthase inhibitor.

## 2. Materials and methods

### 2.1. Drugs and ELISA kits

L-NAME was procured from Santa Cruz BioTech, located on Finnell Street in Dallas, United States. The ELISA test kits for determining cGMP, eNOS, and insulin concentrations were bought from Elabscience Biotechnology Inc., corporate USA.

### 2.2. Collection and identification of plant

The *Peristrophe bivalvis* leaf was harvested from its natural environment in Ikono Local Government Area of Akwa-Ibom, Nigeria. The leaves were subsequently identified and verified at the Pharmacognosy and Herbal Medicine Department, Faculty of Pharmacy, University of Uyo, Nigeria, and assigned an identification number (UUHO43).

### 2.3. Preparation and extraction of *Peristrophe bivalvis* leaf

The leaves of *Peristrophe bivalvis* were double-rinsed. They were first washed with clean water and then with distilled water. The thoroughly washed leaves were air-dried at room temperature and then pulverized. The extraction of *Peristrophe bivalvis* leaf was done using cold extraction techniques. 1000 g of the pulverized leaf was macerated in 10 liters of distilled water (DW) for 3 days with constant stirring. The mixture was filtered using muslin cloth and then Whatman’s filter paper. The concentration of the filtrate was done using a rotary evaporator at 35°C. The concentrate obtained was freeze-dried. The yield obtained after freeze drying was 130 g at a percentage yield of 13.0%. The extract was labeled appropriately and stored in the refrigerator at 4°C until needed.

### 2.4. Qualitative phytochemical examination and in-vitro antioxidant activity

The phytochemical analysis of the aqueous extract of *Peristrophe bivalvis* leaf was determined as follows: the presence of tannins, alkaloids, glycosides, flavonoids, and sterols was determined as described by Edeoga *etal*. [41]. Saponins, as described by Brunner [42], phenol, terpenoids, resins, and phlobatannins were determined as described by Harborne [43].

Briefly, the test for tannins was done by boiling 0.25g of APB in 10 mL of distilled water in a test tube. This was left to cool and then filtered, and 2 drops of 0.1% ferric chloride were added to 1 mL of the filtrate. A change of colour from transient greenish to black indicated the presence of tannins. The alkaloids test was determined by adding 1% hydrochloric acid to 3 mL of the extract solution prepared above in a water bath. After which, Mayer’s and Wagner’s solution was added. The presence of alkaloids was marked by precipitation and turbidity. The saponins test was carried out by heating 2 g of the extract in 20 mL of distilled water in a water bath. The solution obtained was filtered, and 5 mL of the filtrate was mixed with 2.5 mL of distilled water. This was shaking vigorously, and then 3 drops of olive oil were added. All these were shaking together vigorously. The presence of saponins was indicated by the formation of an emulsion. The test for glycosides was done by adding 2 mL of glacial acetic acid containing 2 drops of 2% ferric chloride to 5 mL of the extract solution, and this was mixed together. After which, 2 mL of concentrated sulfuric acid (H_2_SO_4_) was added. The presence of glycosides was indicated by the formation of a brown ring at the interface and a violet ring below the brown ring. The flavonoids test was evaluated by adding 5 mL of ethyl acetate to 1 g of APB, and this was heated in a water bath for 3 minutes. The solution was filtered, and 4 mL of the filtrate was mixed with 1 mL of diluted ammonia solution. The presence of flavonoids was shown by the appearance of a yellow colour, which disappeared after a few minutes. Phenol assessment was carried out by adding 4 drops of ferric chloride solution to 2 mL of ABP solution, which was gently mixed together. The presence of phenols was shown by the formation of a bluish-black colour. Terpenoid was evaluated by vigoriously mixing 4 mL of the extract solution with 2 mL of chloroform in a test tube, and then 2 mL of concentrated sulfuric acid was added through the side of the test tube to the mixture. The appearance of a reddish brown colour at the interfaces showed the presence of terpenoid. The presence of resins was determined by adding a few drops of acetic anhydride solution to 1 mL of the extract solution, and these were mixed together, and 1 mL of concentrated H_2_SO_4_ was added to the mixture. The appearance of an orange colour showed the presence of resins. The test for phlobatannins was done by adding a few drops of hydrochloric acid solution to 1 mL of the APB solution; these were mixed together and then heated. The appearance of a red precipitate showed phlobatannins presence. The sterols test was determined by adding 2 mL of acetic anhydride to 0.5 g of APB, followed by 2 mL of sulfuric acid. The presence of sterols was shown by the change of colour from violet to green.

The diphenyl-1picryl hydrazyl hydrate (DPPH) assay was carried out based on the principle described by Blois [44]. DPPH radical is reduced when it reacts with a compound that has antioxidant ability. There action of DPPH with an antioxidant causes a conversion to light yellow from strong violet, and the concentration was determined at 517nm. Total antioxidant capacity assessment was done based on the principle described by Prieto etal. [45]. The thiobarbituric acid (TBA) reaction was used to examine the antlipid peroxidation activity of APB using the procedure described by Masao *etal*. [46].

Briefly, the assessment of DPPH was done by preparing different concentrations of APB (10, 5, 2.5, 1.25, 0.625, 0.3125mg/mL). Vitamin C was used as the standard. 1.0 mL of 0.3 mM DPPH in methanol was added to 1mL of each of the concentrations. They were mixed properly and left in the dark for thirty minutes, after which the intensity of each was measured at 517nm.Total antioxidant capacity was evaluated by dissolving 0.2g of APB in 1 mL of distilled water. 0.1 mL of APB and different concentrations of ascorbic acid solution (20, 40, 60, 80, and 100 μg/mL), which act as the standard, were measured into separate test tubes. Then 1mL of the reagent solution (0.6 M sulfuric acid, 28mM sodium phosphate, and 4mM ammonium molybdate) was added to each of them. These were properly mixed together and placed in a water bath at 95°C for ninety minutes. They were left to cool, and their intensities were determined at a wave length of 695nm. The anti-lipid peroxidation activity of APB was assessed by using a rat liver homogenate, which was used to prepare a reagent mixture containing 0.1mL of the homogenate and 0.05mL of 0.07M of ferrous sulphate. Different concentrations (0.05 – 0.5mg/mL) of APB were prepared, and 0.5 mL of each of these and 0.05 mL of 1% (weight/volume) ascorbic acid (standard) were measured into separate test tubes. Then, the reagent mixture (0.02 mL) was measured into all the test tubes. They were incubated for one hour at a temperature of 37°C. After which, 0.5 mL of 0.1 N hydrogen chloride, 0.2 mL of 9.8% sodium dodecyl sulphate, 0.9 mL of distilled water, and 2 mL of 0.67% TBA were added sequentially. These were heated for thirty minutes in a water bath (100°C) and allowed to cool. 2.0 mL of butan-1-ol was added to each of them and mixed together. The mixtures were centrifuged for ten minutes at 3000 rpm, and the supernatant was obtained, and the intensity was measured at 532 nm.

### 2.5. Gas chromatography-mass spectroscopy (GC–MS) analysis

The GC-MS analysis of APB was done as described by Rajeswari et al. [47] and Qadir et al. [48]. Briefly, 2 g of APB was dissolved in 4 mL of methanol and spun at 5000 g for 10 min, and then 0.05 mL of the volatile supernatant was transferred to a Varian 450 gas chromatograph (VF-5 MS column). The mass spectra of the components in APB were determined between 50 and 55 min. The chemical compounds present in APB were determined by comparing the retention time and mass spectra data in the National Institute of Standards and Technology (NIST) MS Search 2.0 library.

### 2.6. Experimental animals

For this investigation, male Wistar rats weighing 150–170 g and 10–12 weeks old were used. The rats were maintained in the Animal House of the College of Medicine at the University of Ibadan, Nigeria, after being obtained from the Central Animal House of the university. Prior to the start of the study, the animals were given free access to pellet food and water, as well as a three-week acclimatization phase during which they were exposed to a 12/12 h light and dark cycle at a temperature of 25±3°C. The University of Ibadan’s Animal Ethics Committee approved the study’s procedure (UI-ACUREC/2017/058), and handling and care of the rats were conducted in accordance with their guidelines.

### 2.7. Study design

**Fig 1:**
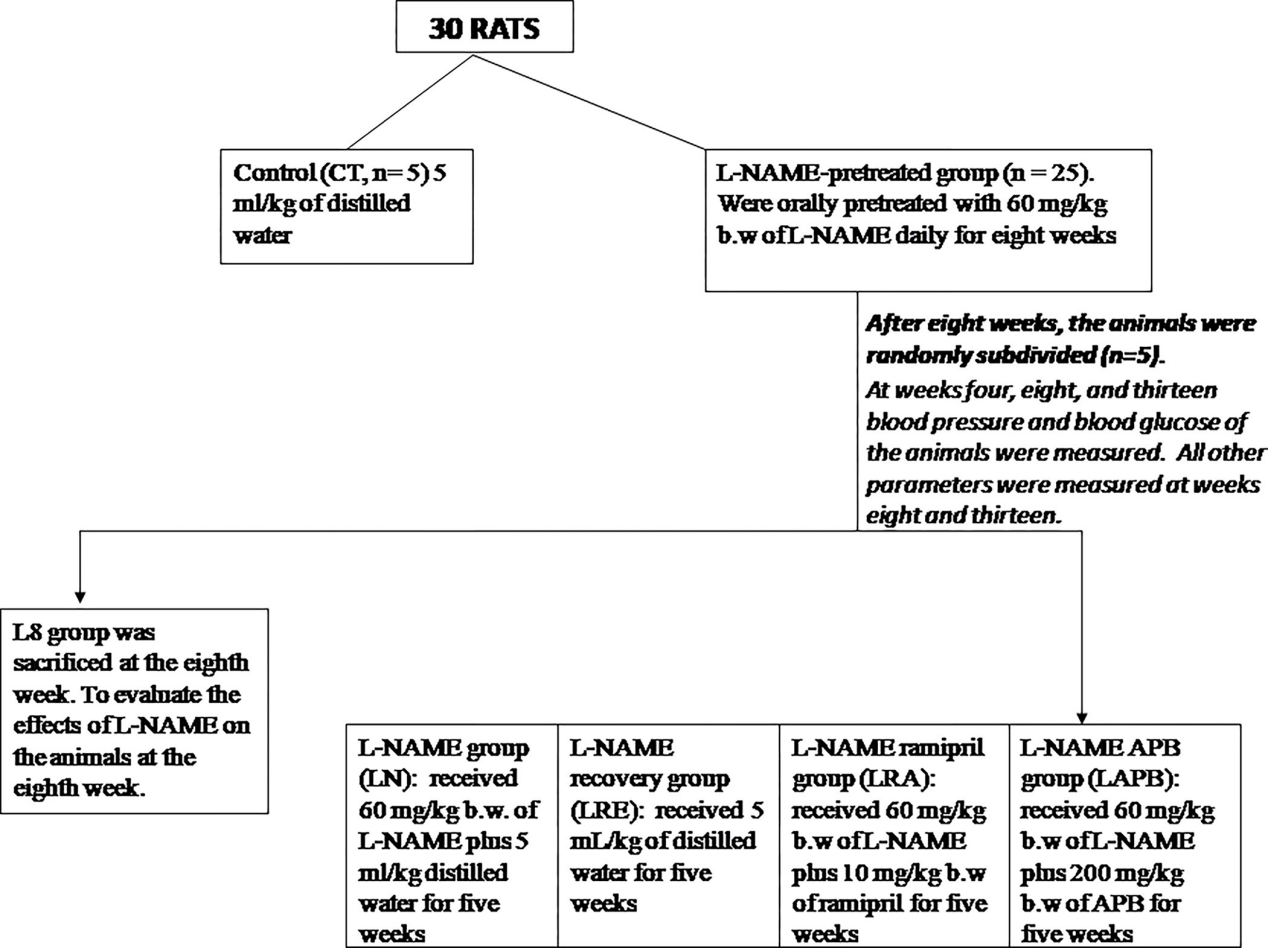
Chart 1. Chart showing the grouping of the animals.

The study used thirty rats, which were unsystematically assigned into two groups: the control group (n = 5) and the L-NAME-pretreated group (n = 25). The control group (CT) was orally gavaged with 5 ml/kg of distilled water (DW) all through the study, while the L-NAME-pretreated group was orally gavaged with 60 mg/kg b.w of L-NAME daily for four weeks. The blood glucose and blood pressure of all the animals were measured at the fourth week, after which the animals in the L-NAME pretreated group were further pretreated with 60 mg/kg of L-NAME for another four weeks. In all, the animals in the L-NAME-pretreated group were pretreated with L-NAME for eight weeks. At the eighth week, the blood glucose and blood pressure of all the rats was measured, and the animals in L-NAME-pretreated group were randomly subdivided into groups as illustrated below (Fig 1). Treatment was done for five weeks, making the duration of the whole procedure 13 weeks. L-NAME and APB were separately dissolved in distilled water before they were administered. The dose for APB (200 mg/kg b.w.) was selected based on our previous study [36].

### 2.8. Measurement of blood pressure and blood glucose

Blood glucose and blood pressure were measured in conscious rats. The blood glucose was determined by the oxidase method using the Accu-Chek Glucometer (Roche Diabetes Care GmbH, Sandhofer Strasse, Mannheim, Germany). The blood pressure was measured by the occlusion tail-cuff technique with the CODA Kent Scientific blood pressure setup. Before the commencement of the study, the animals were trained for one week to get accustomed to the blood pressure set. The rats were put in a restricting device, which was placed on a warm platform (35°C). A medium-sized occlusion cuff and VPR cuff were fixed around the tails of the animals. These cuffs detect the pulsations of the artery. Before the measurement commenced, an acclimatisation period of fifteen minutes was observed. Five acclimation cycles, followed by fifteen actual cycles, were performed by set. The mean of all the measurements (minimum of 3) accepted by the CODA setting, excluding the acclimation cycles and values that are considered outliers (extremely high and low values) for each rat, was taken as the blood pressure.

### 2.9. Blood sample collection

The blood sample was obtained from anesthetized (0.1 ml/100 g b.w. ketamine/xylazine IP.) rats by cardiac puncture into a plain bottle for the analysis of NO, cGMP, eNOS, and insulin levels. Euthanizing the rats was done by administering an overdose of anesthesia. The blood samples were spun at 2500 rpm for ten minutes, and the serum was collected and stored at -20°C until use.

### 2.10. Serum lipid profile and atherogenic ratios assessment

Lipid profile was evaluated as described by Ojiako and colleagues [49]. High-density lipoprotein cholesterol (HDL-C), triglycerides (TG), and total cholesterol (TC) were measured using commercial kits (Fortress Diagnostics Limited, Antrim, UK). Briefly, triglyceride examination is based on theprinciple of the enzymatic colorimetric test. 10μL of the sample and standard were measured into separate test tubes. 1 mL of working solution was added to each test tube. These were incubated at 37°C for 5 minutes, and the intensity was measured at 500 nm and used to estimate the triglyceride level of the sample. Total cholesterol was determined by measuring 0.01 mL of distilled water into a test tube labeled as blank. 0.01 mL of the sample as well as he standard were measured into appropriately labelled test tubes. 1 mL of enzyme reagent was added to all the test tubes; these were incubated for 5 minutes at 37^0^C and the intensity was measured at 500 nm. High density lipoprotein cholesterol (HDL-C) was determinedby adding 0.1 mL of LDL precipitant to 0.2 mL of the sample, these were mixed properly, and the mixture was kept at 25°C for 10 minutes. After which, it was centrifuged at 4000 revolutions for 10 minutes. The supernatant was carefully collected, and the intensity was measured at 500 nm.

The low density lipoprotein cholesterol (LDL-C) was calculated: LDL-C =TC– HDL-C– (TG/5) in mg/dL. Very low density lipoprotein cholesterol (VLDL-C) was calculated by the formula: VLDL-C = TG/5 in mg/dL. The atherogenic ratios were calculated using the following formulas: Atherogenic index of plasma (AI) = log of [TG/HDL-C]; atherogenic coefficient (AC) = (TC - HDL-C)÷ HDL-C; cardiac risk ratio (CRR) = TC÷HDL-C.

### 2.11. Measurement of serum nitric oxide level and serum cGMP, eNOS and insulin

Nitric oxide concentration was measured by the spectrophotometry technique [50]. NO concentration is estimated indirectly by estimating the intensity of nitrite. Nitrite is the byproduct of NO oxidation in the plasma. Briefly, 0.1 mL of Griess reagent, 0.3 mL of the sample, and 2.6 mL of deionized H_2_O were measured into a cuvette and incubated at 25°C for 30 minutes. The blank contained 0.1 mL of Griess reagent and 2.9 mL of deionized H_2_O, while the standard was made with different concentrations of sodium nitrite solution. The intensity was measured at 548nm. A graph of intensity against nitrite level was plotted. The nitrite concentration equivalent to the intensity of the tested samples was determined from the graph. The corresponding concentration is the amount of nitrite in the tested sample. cGMP, eNOS and insulin levels were estimated using ELISA assay kits (Elabscience Biotechnology Inc., corporate, USA). The procedures were done according to the instructions of the manufacturers. Two replicates were done and the average was taken as the result.

### 2.12. Statistical analysis

The data represent the mean ± standard deviation (SD) and were statistically analysed by analysis of variance (ANOVA) and post-hoc comparison (Tukey’s test) using GraphPad Prism 8.0, a product of GraphPad Software, Inc. (USA). Prior to ANOVA, the data set distribution and variance were analysed using Shapiro–Wilk and D’Agostino–Pearson Omnibus normality tests. Body weight and blood glucose of the animals were analysed with two-way ANOVA, while the blood pressure, serum lipid profile, insulin level, NO, cyclic guanosine monophosphate, and endothelial nitric oxide synthase levels were analysed with One-way ANOVA. Values of p < 0.05 were considered statistically significant.

## 3. Results

### 3.1. Assessment of aqueous extract of Peristrophe bivalvis

The result showed that APB has tannins, resins, flavonoids, trepenoids, and a moderate quantity of phenol (Table 1). The antioxidant evaluation showed that APB has a low 50% inhibitory capacity (IC50) for DPPH and malondialdehyde (MDA) and a high total antioxidant capacity (Table 2). Table 3 shows the eighteen chemical compounds identified in APB via GC-MS analysis.

**Table 1:** Qualitative phytochemical screening of aqueous extract of *Peristrophe bivalvis* leaf.

S/N	Phytochemicals	Result
**1**	Tannins	++
**2**	Alkaloids	-
**3**	Saponins	-
**4**	Sterols	-
**5**	Glycosides	-
**6**	Flavonoids	++
**7**	Terpenoid	++
**8**	Resins	++
**9**	phlobatannins	-
**10**	Phenols	+

(-): Absent; (+): Weak positive; (+ +): Positive

**Table 2:** In-vitro antioxidant activitiesof aqueous extract of *Peristrophe bivalvis* leaf.

S/N	Variables	Results
**1**	MDA (IC50) (μg/g of sample)	29.9±1.07
**2**	DPPH (IC50)(μg/mL)	9.91±1.18
**3**	TAC (mgAAE/g)	87.3±0.82

DPPH- 2,2-diphenyl-1-picrylhydrazyl, MDA- Malondialdehyde and TAC- total antioxidant capacity.

**Table 3:** Gas chromatography-mass spectroscopy (GC-MS) analysis of aqueous extract of *Peristrophe bivalvis* leaf.

S/N	Retention time (min)	Quantity (%)	Compound name	Chemical formula	Molecular Weight (g/mol)
**1**	5.587	1.605	Indole-2-one,2,3-dihydro-Nhydroxy-4-methoxy-3,3- dimethyl	C_11_H_13_NO_3_	207
**2**	14.639	1.485	2,2,4- trichloro-1, 3- cyclopentenedione	C_5_HCl_3_O_2_	199
**3**	18.042	1.325	Silicic acid diethyl bis(trimethylsilyl) ester	C_10_H_28_O_4_Si_3_	296
**4**	19.743	1.386	Acetic acid, mercapto-methyl ester	C_3_H_6_O_2_S	106
**5**	21.945	1.285	n-Decanoic acid	C_10_H_20_O_2_	172
**6**	22.758	1.654	Benzo [h] quinoline 2 4- dimethyl	C15H13N	207
**7**	23.008	1.327	(R, R) tartaric acid	C4H6O6	150
**8**	23.784	1.584	7,9- dimethyl-7H- 5,6,7,9,11apentaaza-benzo[a] fluorene8,10-dione	C21H17N5O2	371
**9**	25.873	1.255	Quinoline, 2- (1-methyl- 1Himidazol-4-yl)	C13H11N3	209
**10**	27.012	1.248	1,2-Benzisothiazol-3-amine	C7H6N2S	150
**11**	27.343	41.381	Cycloheptasiloxane, tetradecamethyl	C14H42O7Si7	519
**12**	28.232	1.913	2- Ethylacridine	C15H13N	207
**13**	29.458	1.282	3- Phenylfurazan	C8H6N2O	146
**14**	31.910	1.715	4- Nitro-2-methylaniline, N-tertbutyldimethylsilyl	C13H22N2O2Si	266
**15**	32.110	21.035	Cyclooctasiloxane hexadecamethyl	C16H48O8Si8	593
**16**	34.406	1.259	Benzaldehyde, 2-hydroxy-5- nitro	C_7_H_5_NO4	167
**17**	35.075	9.444	Indole, 3- (4-nitrophenylamino)	C_14_H_11_N_3_O_2_	253
**18**	36.113	7.818	Cyclononasiloxane, octadecamethyl	C_18_H_54_O_9_Si_9_	667

### 3.2. Change in body weight

**Fig 2:**
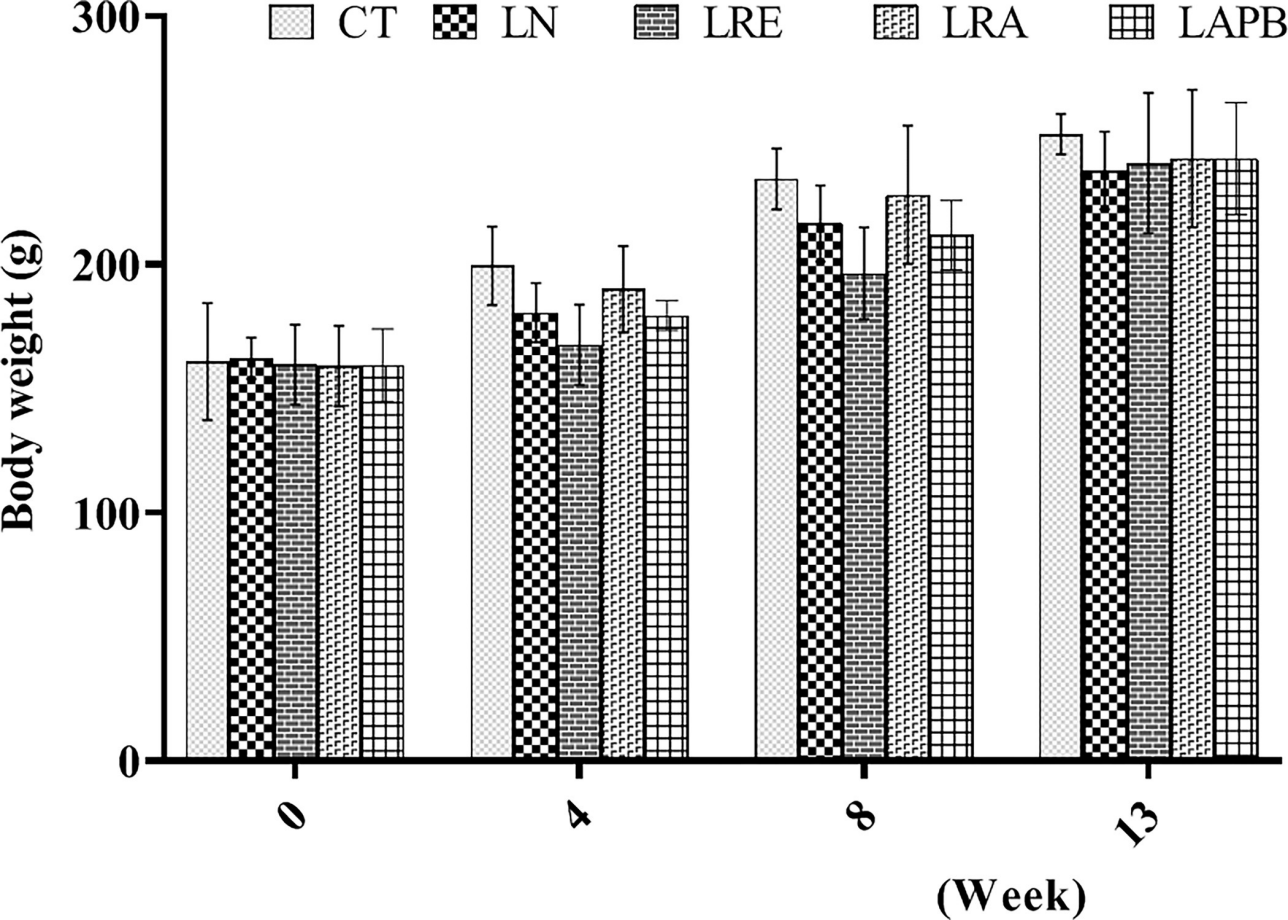
Body weight changes after pretreatment with L-NAME, followed by treatment with APB, ramipril, or distilled water. All the groups except the control group were pretreated with 60 mg/kg of L-NAME. CT –control group received 5 ml/kg distilled water (DW),LN- L-NAME pretreated group that received L-NAME60+ 5 ml/kg DW, LRE – L-NAME pretreated group left to recovery (5 ml/kg DW), LRA - L-NAME pretreated group treated with ramipril (L-NAME60 + 10 mg/kg ramipril), and LAPB – L-NAME pretreated group treated with APB (L-NAME60 + 200 mg/kg APB). Each value represents mean±SD and n=5. APB - Aqueous extract of *Peristrophe bivalvis* leaf and L-NAME- Nitro-L-arginine methyl ester.

There was a progressive change in body weight in all groups. The pretreatment with 60 mg/kg/day of *N*G-nitro-l-arginine methyl ester (L-NAME60) for eight weeks did not significantly change the body weight of the animals compared to the control group. Furthermore, the administration of 200 mg/kg/day of APB did not significantly change their body weight compared to the control and the LN groups (Fig 2).

### 3.3. Blood pressure

Table 4 shows the changes in blood pressure. At the fourth and eighth weeks of the study, systolic blood pressure (SBP), diastolic blood pressure (DBP), and mean arterial pressure (MAP) significantly increased in the L-NAME pretreated group compared to the control group (p< 0.0001 and {MAP: p= 0.0002}). At the end of the study, SBP, DBP, and MAP significantly increased in LN, LRE, and LAPB groups compared to the control group (p< 0.0001, and p= 0.0001 respectively); however, the blood pressure in LRE and LAPB significantly decreased compared to LN (p< 0.0001).

**Table 4:** Changes in blood pressure after pretreatment with L-NAME, followed by treatment with APB, ramipril, or distilled water.

VARIABLES (mm Hg)	GROUPS						
	**CT**	**L4**	**L8**	**LN**	**LRE**	**LRA**	**LAPB**
SBP	124 ±5.00	143 ±5.20^#^	172 ±6.20^#*^	196 ±4.50^#^	152 ±6.40^#*^	132 ±2.50*	148 ±2.30^#*^
DBP	91 ±3.42	111 ±8.02^#^	131 ±5.68^#*^	158 ±6.22^#^	117 ±5.17^#*^	100 ±2.49*	110 ±6.69^#*^
MAP	102 ±3.58	122 ±6.16^#^	144 ±5.60^#*^	171 ±4.30^#^	128 ±5.46^#^	110 ±1.92*	123 ±4.66^#*^

All the groups except the control group were pretreated with 60 mg/kg of L-NAME. CT –control group received 5 ml/kg distilled water (DW), L4 and L8- blood pressure measured at weeks 4 and 8 respectively, LN- L-NAME pretreated group that received L-NAME60+ 5 ml/kg DW, LRE – L-NAME pretreated group left to recovery (5 ml/kg DW), LRA - L-NAME pretreated group treated with ramipril (L-NAME60 + 10 mg/kg ramipril), and LAPB – L-NAME pretreated group treated with APB (L-NAME60 + 200 mg/kg APB). Each value represents mean±SD. n=5, ^#^ = p< 0.05 compared to CT and * = p< 0.05 compared to LN. SBP- Systolic blood pressure, DBP- Diastolic blood pressure, MAP- Mean arterial pressure, APB-Aqueous extract of *Peristrophe bivalvis* leaf and L-NAME-Nitro-L-arginine methyl ester.

### 3.4. Fasting blood glucose level (FBG) and insulin concentration

**Fig 3:**
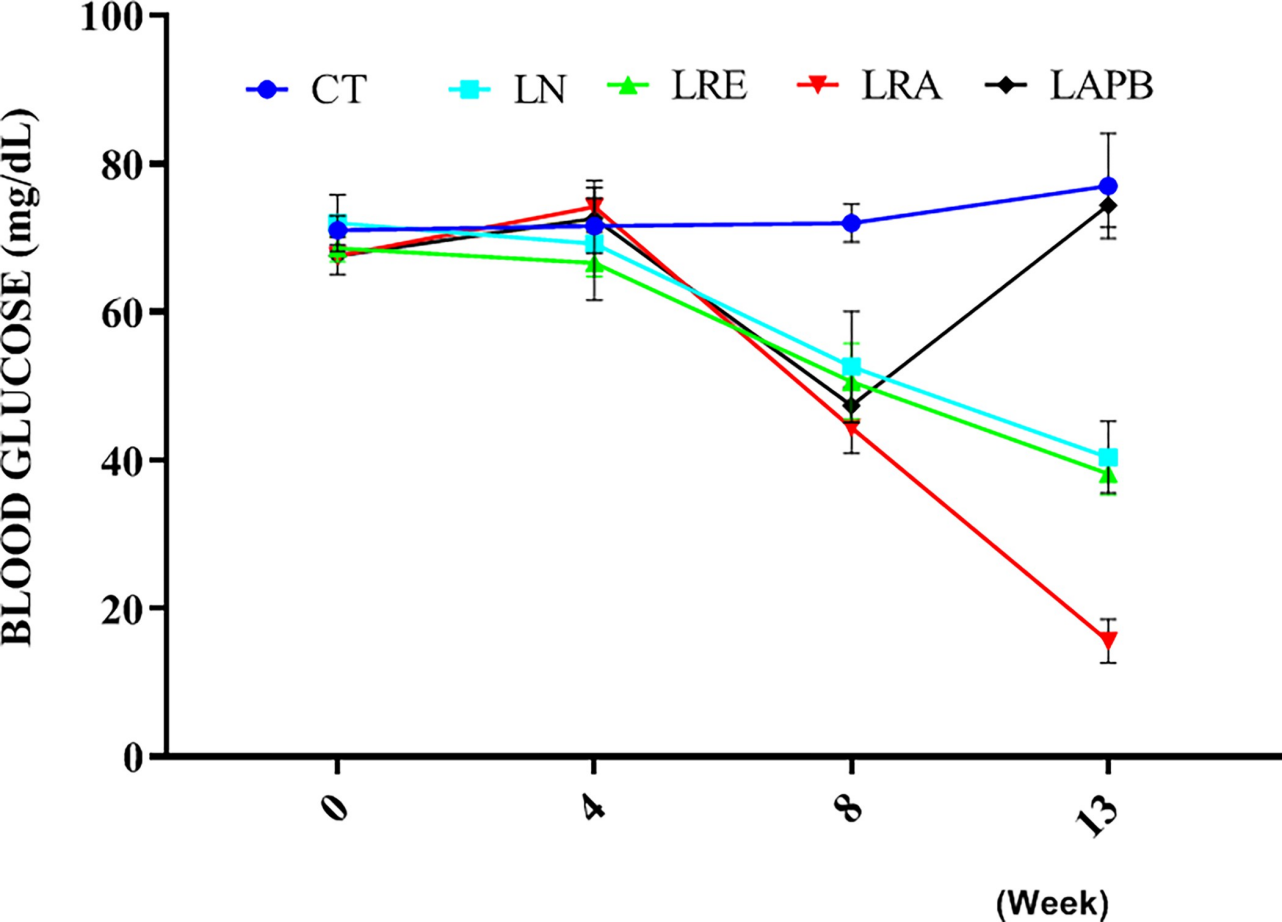
Changes in fasting blood glucose level following L-NAME pretreatment and subsequent treatment with APB, ramipril, or distilled water. All the groups except the control group were pretreated with 60 mg/kg of L-NAME. CT –control group received 5 ml/kg distilled water (DW), LN- L-NAME pretreated group that received L-NAME60+ 5 ml/kg DW, LRE – L-NAME pretreated group left to recovery (5 ml/kg DW), LRA - L-NAME pretreated group treated with ramipril (L-NAME60 + 10 mg/kg ramipril), and LAPB – L-NAME pretreated group treated with APB (L-NAME60 + 200 mg/kg APB). Each value represents mean±SD, n=5, ^#^ = p< 0.05 compared to CT, * = p< 0.05 compared to LN, ^a^ = P< 0.05 compared to LRE, and ^b^ = p<0.05 compared to LRA. APB-Aqueous extract of *Peristrophe bivalvis* leaf and L-NAME-Nitro-L-arginine methyl ester.

**Fig 4:**
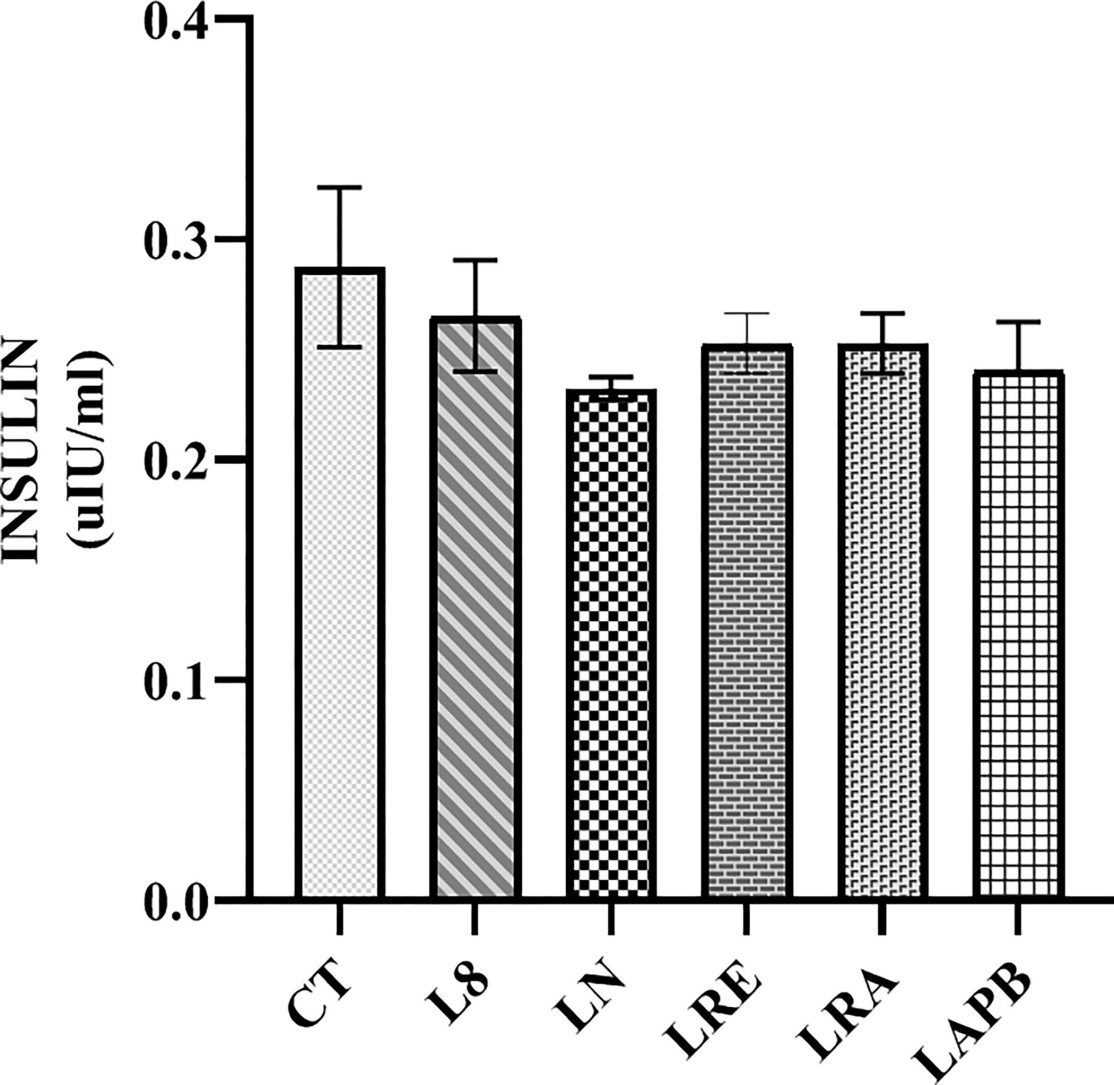
Changes in serum insulin concentration after pretreatment with L-NAME, followed by treatment with APB, ramipril, or distilled water. All the groups except the control group were pretreated with 60 mg/kg of L-NAME. CT –control group received 5 ml/kgdistilled water (DW), L8- L-NAME pretreated group sacrificed the 8^th^ week, LN- L-NAME pretreated group that received L-NAME60+ 5 ml/kg DW, LRE – L-NAME pretreated group left to recovery (5 ml/kg DW), LRA - L-NAME pretreated group treated with ramipril (L-NAME60 + 10 mg/kg ramipril), and LAPB – L-NAME pretreated group treated with APB (L-NAME60 + 200 mg/kg APB). Each value represents mean±SD and n=5. APB-Aqueous extract of *Peristrophe bivalvis* leaf and L-NAME-Nitro-L-arginine methyl ester.

The fasting blood glucose recorded at the fourth week of the experiment in the L-NAME pretreated group was not significantly different from that of the control. However, at the eighth week of the experiment, the fasting blood glucose of the L-NAME-pretreated groups significantly decreased compared to the control group (p<0.0001). Fasting blood glucose decreased further in the LN, LRE and LRA groups compared to the control group (p<0.0001), but the LAPB group showed a significant increase in fasting blood glucose level compared to the LN, LRE and LRA groups (p<0.0001) at the thirteenth week of the experiment (Fig 3). There was no significant change in insulin level in all the tested groups compared to the control group (Fig 4).

### 3.5. Serum lipids levels

The results showed a significant increase in TC, TG, LDL-C, VLDL and, AI, AC, and CRR but a significant decrease in HDL-C at the eighth week (L8) and in LN group compared to CT (p<0.0001, {L8 TG and VLDL: P= 0.02}). The hypertensive rats treated with APB and ramipril, respectively, as well as the hypertensive rats left to recover, showed a significant decrease in TC, TG, LDL-C, VLDL-C, AI, AC, and CRR, but a significant increase in HDL-C compared to LN (TG& VLDL P= 0.0002, 0.0073, 0.004, respectively; HDL-C P= 0.02, 0.001, 0.0002, respectively; TC, LDL-C, AI, AC & CRR p<0.0001) (Table 5).

**Table 5:** Variations in lipid profile following L-NAME pretreatment and subsequent treatment with APB, ramipril, or distilled water.

**Parameters**	**Groups**							
	**CT**		**L8**		**LN**	**LRE**	**LRA**	**LAPB**
**TG (mg/dL)**		65 ±6.05		84 ±0.01#	99 ±12.96#	70 ±12.96*	78 ±3.81*	77 ±6.49*
**TC (mg/dL)**		157 ±13.86		233 ±9.17#*	291 ±18.57#	200 ±26.83#*	168 ±11.63*	168 ±31.32*
**LDL-C (mg/dL)**		107 ±16.56		195 ±7.76#*	255 ±15.71#	160 ±30.00#*	124 ±7.17*	123 ±26.13*
**VLDL-C (mg/dL)**		13 ±1.21		17 ±0.00#	20 ±2.59#	14 ±2.59*	16 ±0.76*	15 ±1.30*
**HDL-C** **(mg/dL)**		36 ±6.49		21 ±2.21#	16 ±2.69#	25 ±2.92#*	28 ±4.71*	30 ±4.25*
**AI**		0.26 ±0.10		0.60 ±0.04#*	0.80 ±0.07#	0.44 ±0.07#*	0.45 ±0.06#*	0.41 ±0.03#*
**AC**		3.4 ±0.44		10 ±0.86#*	17 ±2.03#	7.0 ±1.65#*	5.1 ±0.98*	4.5 ±0.46*
**CRR**		4.4 ±0.44		11.0 ±0.87#*	18.0 ±2.03#	8.0 ±1.65#*	6.1 ±0.98*	5.6 ±0.46*

All the groups except the control group were pretreated with 60 mg/kg of L-NAME. CT –control group received 5 ml/kg distilled water (DW), L8- L-NAME pretreated group sacrificed the 8^th^ week, LN- L-NAME pretreated group that received L-NAME60+ 5 ml/kg DW, LRE – L-NAME pretreated group left to recovery (5 ml/kg DW), LRA - L-NAME pretreated group treated with ramipril (L-NAME60 + 10 mg/kg ramipril), and LAPB – L-NAME pretreated group treated with APB (L-NAME60 + 200 mg/kg APB). Each value represents mean±SD, n=5, # = p< 0.05 compared to CT, and

* = p< 0.05 compared to LN. TG- triglyceride, TC- total cholesterol, LDL-C- low density lipoprotein cholesterol, VLDL-C- very low density lipoprotein cholesterol, HDL-C- high density lipoprotein cholesterol, AI- atherogenic index, AC- atherogenic coefficient and CRR- cardiac risk ratio. APB-Aqueous extract of *Peristrophe bivalvis* leaf and L-NAME-Nitro-L-arginine methy lester.

### 3.6. Serum nitric oxide level

**Fig 5:**
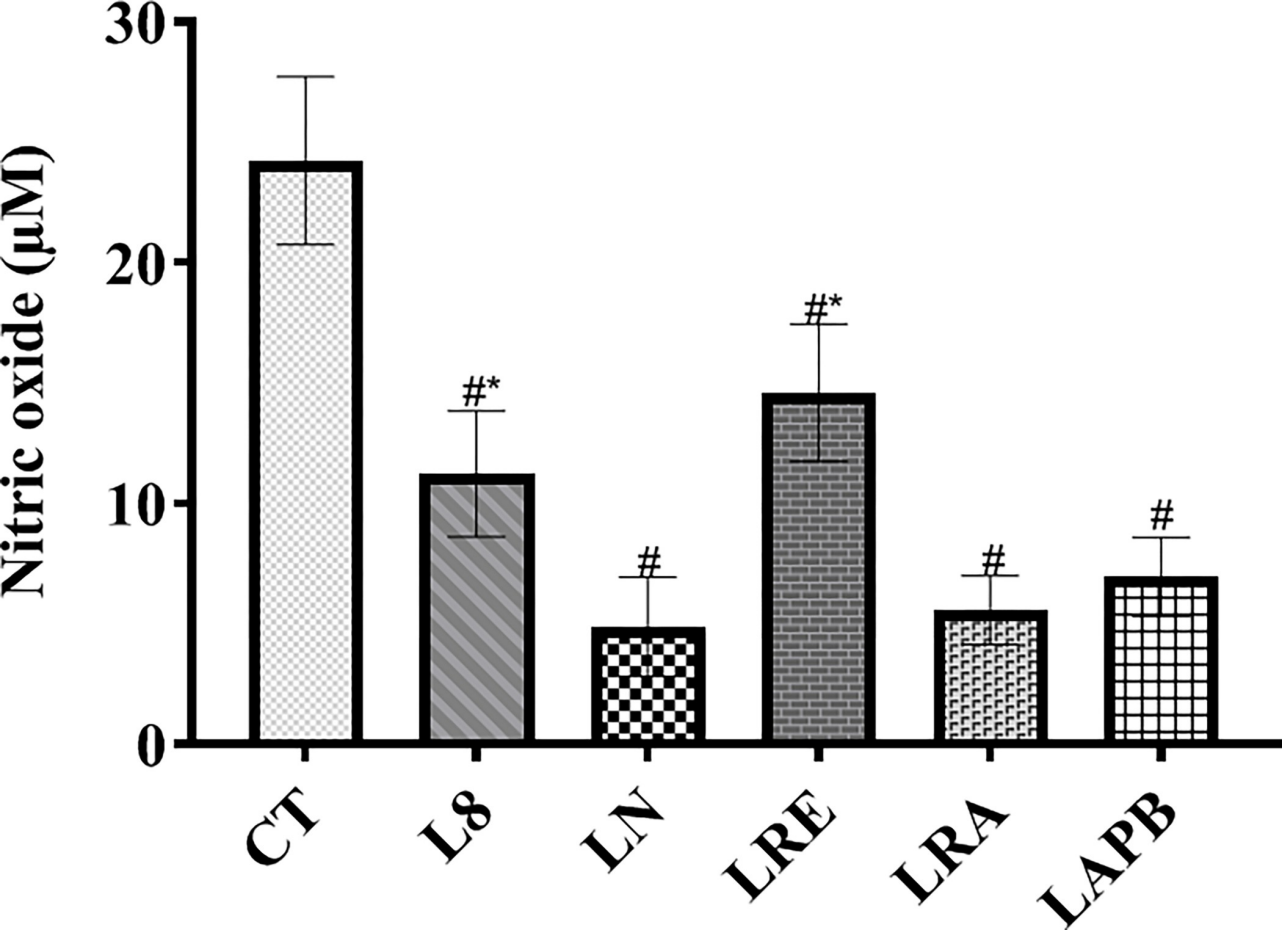
Changes in serum nitric oxide concentration after pretreatment with L-NAME, followed by treatment with APB, ramipril, or distilled water. All the groups except the control group were pretreated with 60 mg/kg of L-NAME. CT –control group received 5 ml/kg distilled water (DW), L8- L-NAME pretreated group sacrificed the 8^th^ week, LN- L-NAME pretreated group that received L-NAME60+ 5 ml/kg DW, LRE – L-NAME pretreated group left to recovery (5 ml/kg DW), LRA - L-NAME pretreated group treated with ramipril (L-NAME60 + 10 mg/kg ramipril), and LAPB – L-NAME pretreated group treated with APB (L-NAME60 + 200 mg/kg APB). Each value represents mean±SD, n=5, ^#^ = p< 0.05 compared to CT, and * = p< 0.05 compared to LN. APB-Aqueous extract of *Peristrophe bivalvis* leaf and L-NAME-Nitro-L-arginine methy lester.

Fig 5 shows the changes in nitric oxide level. A significant decrease was observed in nitric oxide level in the L-NAME-pre-treated group (L8) compared to the control group (p<0.0001), and at the end of the experiment, nitric oxide level was significantly decreased in the LN, LRE, LRA, and LAPB groups compared to the control (p<0.0001). However, in the LRE group, the nitric oxide level was significantly increased compared to the LN group (p<0.0001).

### 3.7. Serum cyclic guanosine monophosphate (cGMP) level

**Fig 6:**
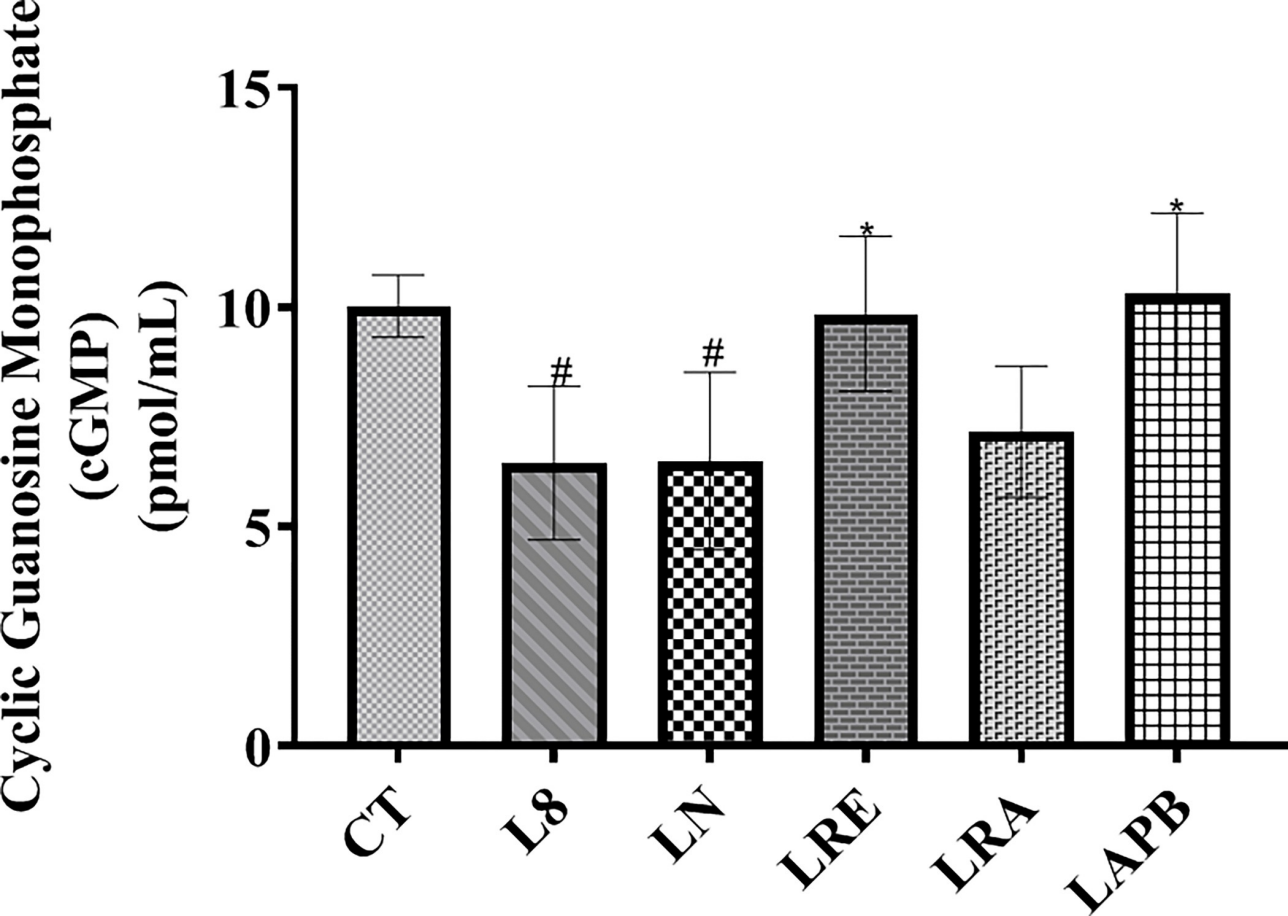
Variations in serum cyclic guanosine monophosphate activity (cGMP) following L-NAME pretreatment and subsequent treatment with APB, ramipril, or distilled water. All the groups except the control group were pretreated with 60 mg/kg of L-NAME. CT –control group received 5 ml/kg distilled water (DW), L8- L-NAME pretreated group sacrificed the 8^th^ week, LN- L-NAME pretreated group that received L-NAME60+ 5 ml/kg DW, LRE – L-NAME pretreated group left to recovery (5 ml/kg DW), LRA - L-NAME pretreated group treated with ramipril (L-NAME60 + 10 mg/kg ramipril), and LAPB – L-NAME pretreated group treated with APB (L-NAME60 + 200 mg/kg APB). Each value represents mean±SD, n=5, ^#^ = p< 0.05 compared to CT, and * = p< 0.05 compared to LN.APB-Aqueous extract of *Peristrophe bivalvis* leaf and L-NAME-Nitro-L-arginine methyl ester.

The study showed a significant decrease in cGMP level in the L8 and LN groups compared to the control group (p= 0.024 and 0.027, respectively), but a significant increase was observed in the LRE and LAPB groups compared to the LN group (p= 0.04 and 0.02, respectively) (Fig 6).

### 3.8. Serum endothelial nitric oxide synthase (eNOS) concentration

**Fig 7:**
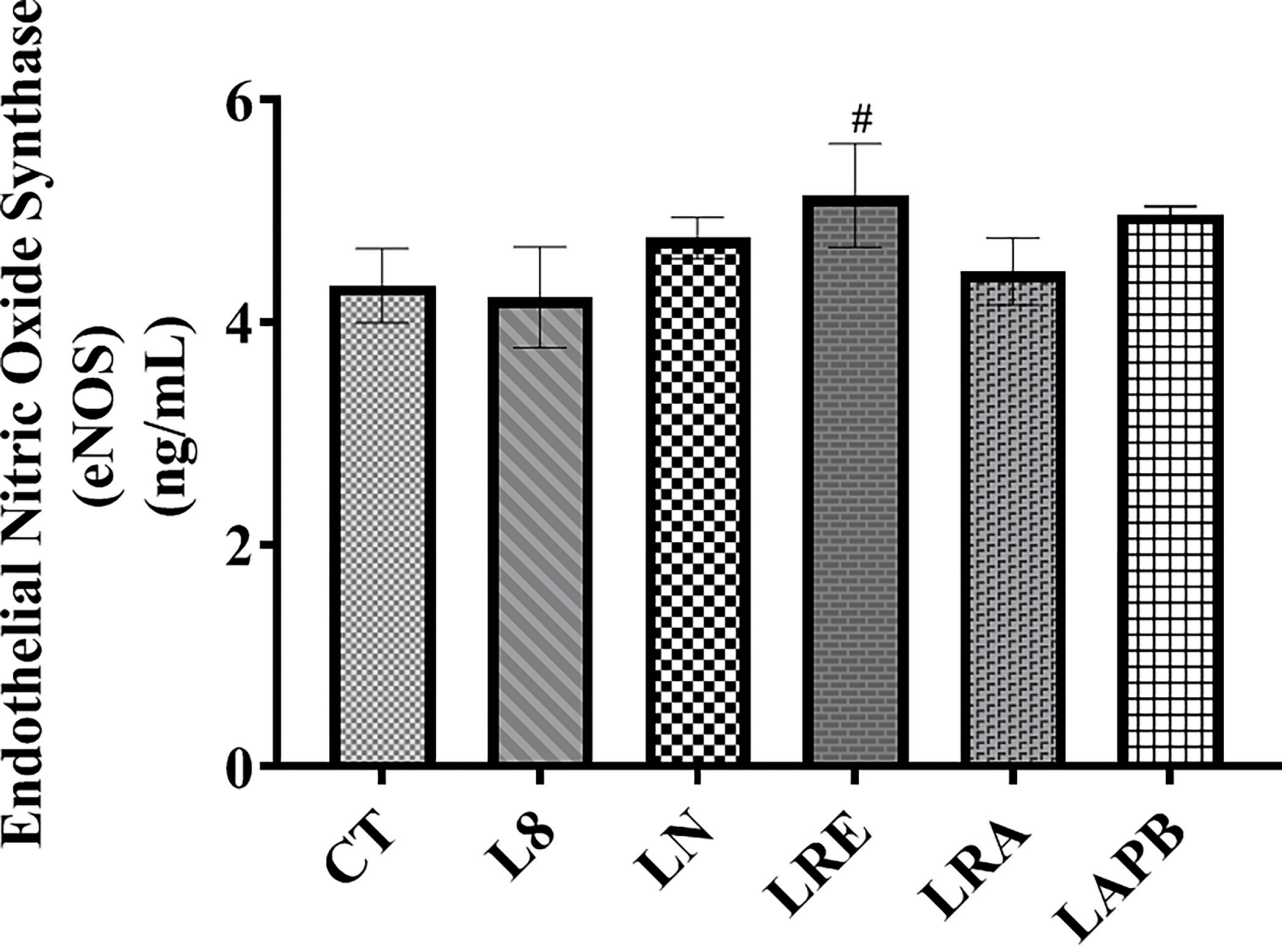
Changes in serum endothelial nitric oxide synthase (eNOS) concentration after pretreatment with L-NAME, followed by treatment with APB, ramipril, or distilled water. All the groups except the control group were pretreated with 60 mg/kg of L-NAME. CT –control group received 5 ml/kg distilled water (DW), L8- L-NAME pretreated group sacrificed the 8^th^ week, LN- L-NAME pretreated group that received L-NAME60+ 5 ml/kg DW, LRE – L-NAME pretreated group left to recovery (5 ml/kg DW), LRA - L-NAME pretreated group treated with ramipril (L-NAME60 + 10 mg/kg ramipril), and LAPB – L-NAME pretreated group treated with APB (L-NAME60 + 200 mg/kg APB). Each value represents mean±SD, n=5, ^#^ = p< 0.05 compared to CT. APB-Aqueous extract of *Peristrophe bivalvis* leaf and L-NAME-Nitro-L-arginine methyl ester.

Fig 7 shows the changes in eNOS level. The study showed no significant changes in eNOS concentration in all the tested groups; however, in the LRE group, a significant increase in eNOS level was observed compared to the control group (p= 0.009).

## 4. Discussion

The influence of nitric oxide (NO) on glucose metabolism has been documented, although not congruent. Some studies have reported that NO facilitates the uptake of glucose by skeletal muscle [23, 21], while others have demonstrated that NO does not modulate glucose uptake by the skeletal muscle [19, 20]. In our lab, we demonstrated that the blockage of NOS resulted in a decrease in fasting blood glucose level [51]. Similar to our result, Bryan and colleagues [30] demonstrated that NO donor efficiently increased blood glucose level, which was effectively reduced by the administration of L-NAME. Likewise, antihypertensive agents have been documented to modulate the metabolism of glucose. *Peristrophe bivalvis* has been reported to exhibit antihypertensive potential [34, 36]. The study assessed the impact of *Peristrophe bivalvis* leaf (aqueous extract) on fasting blood glucose level in rats pretreated with L-NAME, a non-selective nitric oxide synthase inhibitor. The study pretreated all the rats with L-NAME except for those in the control group for eight weeks. After pretreatment with L-NAME, the administration of L-NAME was discontinued in rats assigned to the recovery group, but the administration of L-NAME continued in the rats assigned to the L-NAME and L-NAME-APB groups. Our previous study’s results [36], which showed that the rats given L-NAME completely recovered from the effects of the drug once L-NAME administration was stopped, served as the basis for the continuous administration of L-NAME in this study.

The results showed a progressive increase in body weight in all the animals. The progressive body weight changes throughout the course of the experiment were not significantly different in all the groups. This observation negates our hypothesis, which was based on previous reports by Joost and Tschop [52]. They explained that nitric oxide modulates energy balance in the body and that a reduction in nitric oxide bioavailability reduces food intake and body weight. Buttressing this, Salami et al. [53] reported a decrease in body weight in rats administered with nitric oxide synthase inhibitor. However, the study by Bernátová and associates [54] disagrees with this assertion. Their findings showed no significant body weight changes in rats treated with L-NAME compared to those in the control group. We can infer from our results that a decrease in nitric oxide bioavailability probably has no effect on energy balance.

There was no significant change in fasting blood glucose level (FBG) at the fourth week of the study; however, at the eighth week, a significant decrease was observed in the L-NAME pretreated group. At the thirteenth week, FBG decreased further in the L-NAME group. This observed effect of L-NAME showed that the influence of L-NAME on FBG is time-dependent. Furthermore, in the recovery group, FBG decreased further compared to the control group; this implied that the L-NAME effect on FBG might be irreversible. However, in the L-NAME-APB group, FBG was restored to a normal level. The glycemic effect of *Peristrophe bivalvis* has not yet been reported, so our study is the first to report its glycemic effect. In the present study, APB showed a restorative effect on FBG. The blood glucose of the rats was monitored from week 0 to week 13, and the result showed that the FBG of the rats in the LAPB group was restored to the level recorded at weeks 0 and 4.

The glycemic effect of APB in this study is independent of insulin; this is inferred because the study recorded no significant change in insulin level in all the tested groups compared to the control. However, its glycemic effect might be linked to NO. Nitric oxide donor has been reported to increase blood glucose level [30]. Although the study recorded a decrease in nitric oxide level in the LAPB group, a significant increase was observed in cGMP level in the rats treated with APB. Nitric oxide acts through a second messenger system by activating soluble guanylyl cyclase. This converts GTP to cGMP, which activates cGMP-dependent protein kinase, and this protein kinase brings about the nitric oxide physiological effects. Aside from nitric oxide, the other components of the NO-signaling circuit are necessary for the initiation of the physiological effects of nitric oxide, and the activation of any of these components can produce NO-induced physiological changes [12]. We can deduce that APB acted independently of NO to stimulate an increase in cGMP levels. This observed effect of APB on cGMP might be that APB has the ability to prevent the breakdown of cGMP by inhibiting phosphodiesterase or activating guanylyl cyclase directly to promote the synthesis of cGMP, causing the recorded increase in cGMP. The increased cGMP activity then caused the blood glucose level to rise to a normal level in the LAPB group.

Nevertheless, this same claim cannot be upheld in the recovery group. The recovery group showed an increase in nitric oxide, eNOS, and cGMP levels, but the FBG was low. This implies that NO might not be responsible for the change in FBG. Glucagon has been documented as one of the mechanisms via which L-NAME modulates blood glucose level. Bryan and coworkers [30] suggested in their study that the hypoglycemic effect of L-NAME might be through the inhibition of glucagon. Buttressing this assertion, studies have reported that nitric oxide synthase inhibitor block the release of glucagon [55, 56].

NO has been reported to modulate the metabolism of lipids and has been documented to activate the transcription factor for low-density lipoprotein cholesterol (LDL-C) receptors and thereby facilitates the removal of LDL-C from the blood into the hepatic cells [31]. The results of the rats in the L-NAME group showed an increase in triglyceride (TG), total cholesterol (TC), LDL-C, and very LDL-C (VLDL-C) levels and a decrease in high-density lipoprotein cholesterol (HDL-C) level. Similarly, Goudarz et al. [32] reported elevated levels of TG, TC, LDL-C, and VLDL-C and a decrease in HDL-C level in animals administered L-NAME. High plasma levels of TC and TG are major risk factors for cardiovascular diseases, and low plasma HDL-C concentration is a key factor predisposing to atherosclerosis and its associated cardiovascular consequences. The L-NAME pretreated rats treated with APB showed a decrease in TC, TG, LDL-C, and VLDL-C and an increase in HDL-C level. The antilipidemic effect of APB might be a result of the increased cGMP level. Atherogenic ratios, which include atherogenic coefficient (AC), cardiac risk ratio (CRR), and atherogenic index of plasma (AIP), act as indicators that show the plasma cholesterol levels that tend towards atherogenic hyperlipidemia. The study recorded low atherogenic ratios in the L-NAME pretreated APB group, and this shows that APB has anti-atherogenic potential.

## 5. Conclusion

The findings of the study showed that APB corrected the elevated lipid levels and low blood glucose level caused by the administration of NG-Nitro-L-arginine methyl ester, and these effects might be via the activation of cyclic guanosine monophosphate. This study showed that APB has a positive impact on lipid and glucose metabolism.

### 5.1. Limitations of the study

The effects of Peritrophe bivalvis leaf on blood glucose and lipid profiles have not been the subject of many previous investigations, making it difficult to compare the results of this study to previous findings. In the present study, the rats given Peristrophe bivalvis leaf extract showed a drop in nitric oxide level but an increase in cyclic guanosine monophosphate. This study could explain the possible cause of this. Furthermore, the study concluded that the mechanism through which Peristrophe bivalvis leaf modulates blood glucose and lipid levels is via the activation of cyclic guanosine monophosphate. However, the activation of cyclic guanosine monophosphate might not be the only route through which Peristrophe bivalvis leaf modulates blood glucose and lipid levels. In addition, the recovery group recorded an increase in nitric oxide, cyclic guanosine monophosphate, and endothelial nitric oxide synthase levels but showed a decrease in blood glucose level. In the current study, the dosage of Peristrophe bivalvis leaf extract used cannot be directly extrapolated to humans. Despite these limitations, the study demonstrated that the aqueous extract of Peristrophe bivalvis leaf exhibited promising results that could be highly beneficial in medicine.
